# Coexistence of Mucormycosis and Granulomatosis With Polyangiitis: A Diagnostic and Therapeutic Challenge

**DOI:** 10.7759/cureus.25124

**Published:** 2022-05-18

**Authors:** Koushik Sanku, Dima Youssef

**Affiliations:** 1 Internal Medicine, East Tennessee State University - Quillen College of Medicine, Johnson City, USA; 2 Hematology and Oncology, Brooklyn Cancer Care, New York City, USA; 3 Internal Medicine, Gandhi Medical College, Hyderabad, IND; 4 Infectious Diseases, East Tennessee State University - Quillen College of Medicine, Johnson City, USA

**Keywords:** anca-associated vasculitis, fungal rhinosinusitis, opportunistic mycoses, amphotericin b, isavuconazonium sulfate, cresemba, isavuconazole, immunocompetent, anti-neutrophil cytoplasmic antibody, mucormycosis

## Abstract

Mucormycosis is a destructive, necrotizing, and potentially fatal fungal disease that usually affects immunocompromised or diabetic patients. Granulomatosis with polyangiitis (GPA), previously known as Wegener’s granulomatosis is a rare, aseptic necrotizing, granulomatous vasculitis affecting small- to medium-sized vessels, resulting in systemic manifestations. Here, we present a case of a 46-year-old gentleman with overlapping features of mucormycosis and GPA, that was successfully treated with isavuconazole monotherapy.

## Introduction

Mucormycosis is an opportunistic fungal infection caused by class Zygomycetes [1]. It is a destructive, necrotizing, and potentially fatal disease [1]. The diagnosis requires identification of the organism in tissue or by culture [1,2]. The treatment involves aggressive surgical debridement combined with systemic antifungal therapy [2]. This infection usually affects immunocompromised or diabetic patients but lately, mucormycosis in immunocompetent is being increasingly reported [1]. Granulomatosis with polyangiitis (GPA), previously known as Wegener’s granulomatosis is an autoimmune vasculitis affecting mainly the upper and lower respiratory tracts and kidneys which is usually treated with immunosuppressive therapy [2]. Here, we present a case of GPA that was uncovered in an immunocompetent individual being treated for fungal rhinosinusitis.

## Case presentation

A 46-year-old male patient with no significant past medical history presented with a 2-month history of fever, nasal congestion, ear, and facial pain eventually progressing to right-sided facial droop that prompted him to visit the hospital. The patient had no significant family history and his social history was negative for any tobacco, alcohol, or illicit drug use. On examination, his tympanic membrane appeared red, bulging with purulent fluid visible around it. Mastoid tenderness and purulent nasal discharge were present. Labs on admission were significant for leukocytosis and reactive thrombocytosis. His chest X-ray was normal, but a computed tomography (CT) angiogram of the head and neck revealed right-sided oto-mastoiditis along with right posterolateral nasopharyngeal wall thickening. An incidental lung nodule was also found in the left upper lobe on a chest CT scan. He was started on intravenous (IV) fluids, broad-spectrum antibiotics per sepsis protocol, along with a short course of oral dexamethasone for facial paralysis. Otolaryngology was consulted, and the patient underwent a right myringotomy with a pressure equalization tube placement in the right ear. Nasopharyngeal biopsy was done which was negative for carcinoma, or any microbes but showed inflamed granulation tissue with necrosis and lymphoid tissue with T cell predominance. As his symptoms improved, he was discharged on oral clindamycin and levofloxacin with a follow-up visit scheduled in the clinic.

He presented to the hospital again a week after finishing his antibiotic course with complaints of progressively increasing facial pain, right ear pain, and a low-grade fever. The patient also reported persistent nasal congestion causing difficulty breathing through the nose and purulent nasal discharge. On examination, he was febrile and tachycardic. Head and neck exam was significant for sinus tenderness, nasal congestion with some visible darkening of the nasal mucosa, and a purulent nasal discharge.

**Figure 1:**
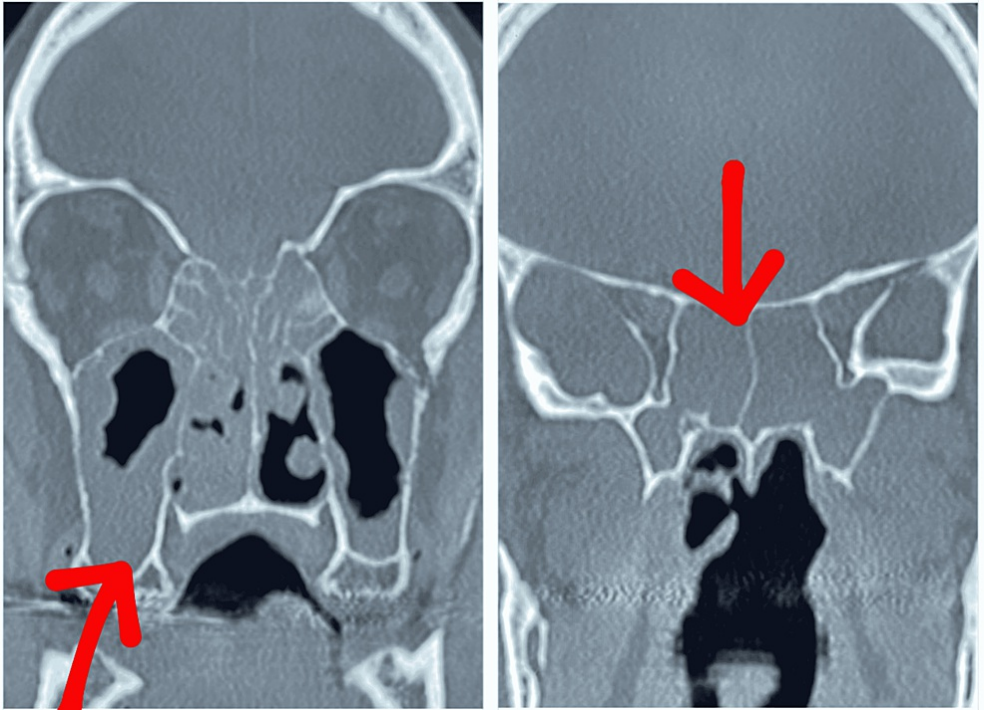
Pansinusitis seen on CT imaging (red arrows)

His blood work revealed neutrophilic leukocytosis with a WBC count of 14,300/µL and a platelet count of 566000/µL. CT scan was positive for pansinusitis (Figure 1) and right oto-mastoiditis. The patient was treated with IV vancomycin and meropenem for sepsis. A repeat biopsy of the nasopharyngeal mass showed necrosis and the presence of fungal organisms with morphology consistent with Zygomycetes on Grocott-Gomori’s methenamine silver (GMS) staining. Thus, vancomycin and meropenem were discontinued and he was started on IV liposomal amphotericin B for sinonasal mucormycosis. However, the patient had worsening transaminitis over the next few days and subsequently developed pruritic rashes over the arms and back due to an allergic reaction to amphotericin B. Treatment regimen was switched to oral isavuconazonium sulfate 372 mg (initial loading with six doses every 8 hours followed by maintenance once daily). Both the rash and transaminitis resolved on cessation of amphotericin B. Functional endoscopic sinus surgery with excision of middle turbinates, total ethmoidectomy, sphenoid and frontal sinusotomy was done to reduce the infection burden.

Further diagnostic workup to determine the patient’s immune status and the nature of nodular lung lesions was done which showed that the patient had an elevated proteinase 3 anti-neutrophil cytoplasmic antibody (pr3-ANCA/c-ANCA) leading us to the diagnosis of GPA. The patient was initiated on immunosuppressive therapy with daily oral prednisone 60 mg. The patient continued to improve and was eventually discharged with a plan to continue oral isavuconazonium sulfate once daily for 6 months, and a tapering course of oral prednisone.

**Figure 2:**
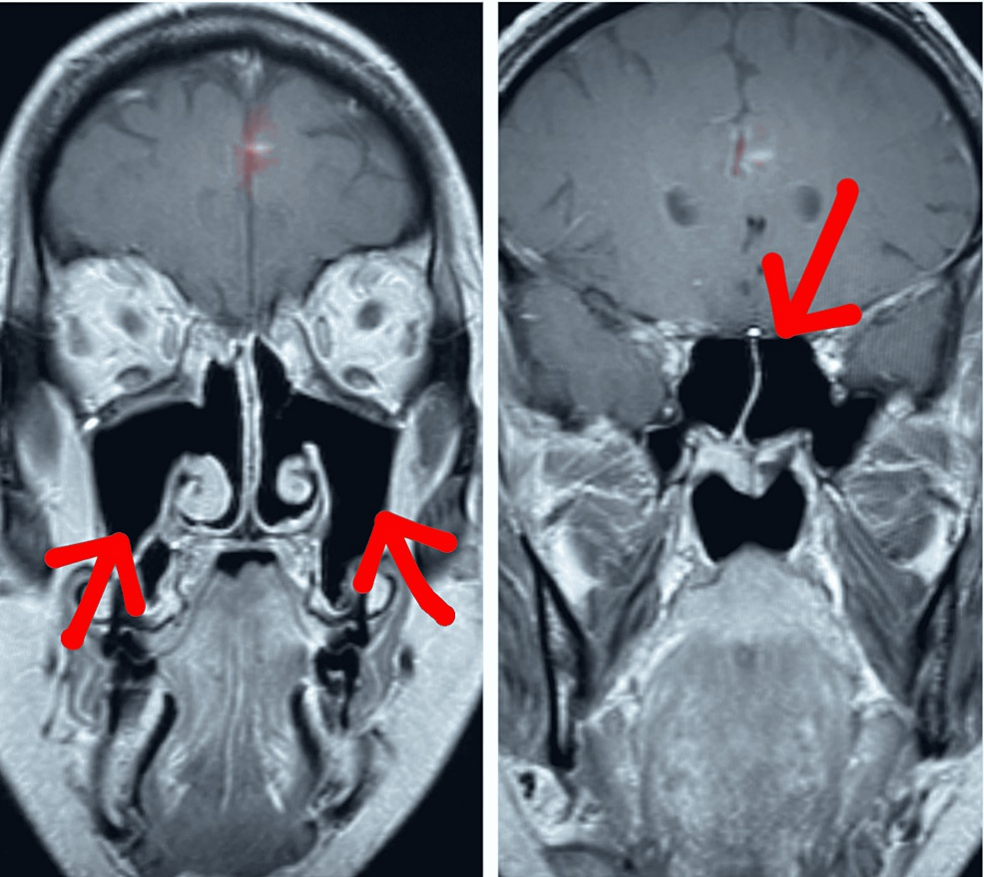
MRI paranasal sinuses post-treatment showing resolution of sinusitis

He was gradually weaned off of steroids, while simultaneously initiating maintenance therapy with weekly parenteral methotrexate and daily oral folate. The patient improved with near-complete resolution of facial palsy. MRI of sinuses post-therapy also showed resolution of infectious/inflammatory processes (Figure 2).

## Discussion

We present a case of GPA presenting in a setting of sinonasal mucormycosis. Mucormycosis is a fatal opportunistic fungal infection mainly seen in presence of risk factors like type 2 diabetes mellitus or immunosuppression (hematologic malignancies, solid organ, or hematopoietic stem-cell transplantation) [1,3]. Infection occurs via the airborne spread of fungal spores which get attached to the nasal mucosa, which can become angio-invasive in a setting of altered immunity resulting in thrombosis and necrosis of nasal turbinate [1,4]. It can be argued that the short course of dexamethasone along with the broad-spectrum antibiotics that the patient received during the initial hospital visit rendered him at risk of opportunistic fungi. However, our case demonstrates the possibility of mucormycosis in an individual without any classic risk factors. Therefore, we believe it is important of having mucormycosis as a differential diagnosis in any individual with an appropriate clinical picture.

GPA is a rare, aseptic necrotizing, granulomatous vasculitis affecting small- to medium-sized vessels and resulting in systemic manifestations. Upper respiratory tracts, lungs, and kidneys are commonly involved [2,5]. The isolated sinonasal disease is present in 25% of cases, presenting with symptoms like nasal congestion, nasal discharge, facial pain or pressure, and anosmia initially [5]. Imaging in such patients may demonstrate non-specific maxillary antral mucosal thickening in the initial stages, while more typical nodular lesions are seen in later stages [5]. As the diagnosis may be missed during the initial stages, the disease often progresses resulting in avascular necrosis of midline sinonasal structures like the nasal septum, turbinates, and anterior ethmoid region [5]. Our patient had similar sinonasal manifestations, and a nodular lung lesion which could be a manifestation of GPA. The overlapping features of GPA and mucormycosis can create a diagnostic and treatment dilemma. Thus, it is important to perform a comprehensive workup to rule out or rule in these diseases before finalizing the management plan. Previous reports of similar cases where immunosuppressive therapy was initiated without addressing the fungal infection led to the dissemination of mucormycosis resulting in poor outcomes [2,3].

Guideline-directed management of mucormycosis-associated rhinosinusitis includes surgical debridement combined with antifungal suppression therapy [2,6]. Intravenous liposomal amphotericin B is the only first-line choice for mucormycosis treatment [6]. Isavuconazole and posaconazole are recently added to the guidelines, however, are still considered second-line therapies [6]. Isavuconazole is a second-generation triazole with activity against several clinically important fungi, including mucor [7]. Phase III VITamin D and OmegA-3 TriaL (VITAL) trial showed that isavuconazole has similar efficacy to amphotericin B in the treatment of patients with mucormycosis [7,8]. Its water-soluble prodrug, isavuconazonium sulfate, is available in both intravenous and oral formulations [7]. Our case provides an example of successful treatment of mucormycosis using isavuconazonium monotherapy as the patient was unable to tolerate amphotericin B. We believe that growing evidence of isavuconazole’s non-inferior efficacy to amphotericin B, combined with the benefits like availability of both oral and parenteral formulations, predictable pharmacokinetics, and minimal toxicity, make it a good treatment choice, especially in patients with renal impairment or allergic reaction to amphotericin B [7,8].

## Conclusions

We present a rare case with both mucormycosis and GPA seen in the same individual. Since these two conditions have overlapping features, a thorough clinical, laboratory, and radiographic workup is needed to differentiate between the two conditions to prevent any misdiagnosis. Isavuconazole monotherapy has proven efficacious in the treatment of mucormycosis and is well tolerated by patients.
